# Age- and sex-dependence of five major elements in the development of human scalp hair

**DOI:** 10.1186/s40824-019-0179-5

**Published:** 2019-12-21

**Authors:** Byeong-Jo Ha, Ga Yun Lee, Il-Hoon Cho, Sangsoo Park

**Affiliations:** 1 Department of Beauty and Cosmetics, College of Bio-convergence, Eulji University, 553 Sanseongdae-ro, Seongnam, Gyeonggi-do 13135 South Korea; 2 Department of Biomedical Laboratory Science, College of Health Science, Eulji University, Seongnam, Gyeonggi-do 13135 South Korea; 3 Department of Biomedical Engineering, College of Bio-convergence, Eulji University, Seongnam, Gyeonggi-do 13135 South Korea

**Keywords:** Mineral element concentration, Scalp hair, Calcium, Magnesium, Age-dependence, Sex-dependence

## Abstract

**Background:**

Human scalp hair is composed of bio-synthesized protein that stores and excretes excess elements from the body. Thus, the concentration of major and trace elements in the hair may provide insight into both the physiology and health status of humans. Monitoring of health status by hair analysis is limited by the uncertainty surrounding natural changes in composition based on age and sex parameters.

**Methods:**

A total of 322 hair samples from subjects aged 0–89 years were collected and analyzed to determine their sulfur, calcium, magnesium, zinc, and copper concentrations by inductively coupled plasma mass spectrometry. The age- and sex-dependence of the concentrations of these elements within scalp hair was evaluated. Age-dependence was analyzed by least squares fitting of the data from young people (up to 25 years old). Sex-dependence was evaluated by comparing the average element concentrations of males and females in each age groups.

**Results:**

The concentration of mineral elements increased with age up to 25 years old. Calcium and magnesium contents were strongly affected by age, whereas the effects were weaker for zinc and copper. In participants over 20 years old, sex and the concentrations of calcium and magnesium were significantly associated. The concentrations of these elements were higher in most of the subgroups of adult women. The concentrations of sulfur, zinc, and copper were not significantly associated with age or sex.

**Conclusions:**

The concentrations of major inorganic elements in scalp hair showed an increasing trend up to 25 years of age, and a strong sex dependence of calcium and magnesium concentrations in the subjects older than 20 years. More research is needed to understand the physiology of calcium and magnesium excretion though scalp hair.

## Background

Human scalp hair is a biological composite material comprised of two main parts: follicles under the dermis and the hair fiber shaft above the skin surface. The hair fiber shaft is mostly composed of keratin proteins, lipids, moisture, and mineral elements that are typically bound to amino acids in proteins or fatty acid groups in lipids [1, 2].

Elemental analysis has shown that hair contains approximately 5% w.t. sulfur. The high sulfur content reflects the high cysteine concentration of scalp hair proteins, enabling disulfide bond formation which confer high tensile strength and elasticity to hair fibers [2].

Calcium, magnesium, zinc, and copper are essential elements in bone, as they help to increase bone strength via interactions with more than 300 enzymes [3]. These elements are also highly abundant in scalp hair. Calcium is the most abundant mineral in the bone and scalp hair.

Calcium ions provided by food pass through the blood, and excess calcium ions are excreted via the renal pathways or deposited in bone and other tissues including blood vessels, hair, and nails. It has been reported that the hair calcium concentration is an indicator of deteriorations in bone metabolism and predictor of coronary artery calcification [4–6]. The relationship between bone mineral density and calcium concentration in the hair is unclear. It is well-established that bone mineral densities increase with age until an organism reaches peak bone mass and then decrease with age [7]. However, the age and sex dependence of major elements in the hair has yet to be established.

Several previous studies have examined the age- and/or sex-dependence of elements found in human scalp hair [8–12] but have shown inconclusive results because of the 1) wide reference ranges of the elements in hair and 2) lack of a large enough sample size to accurately reflect reference ranges within all age groups from newborns to the elderly. The number of samples in previous studies is too small to draw a statistically significant conclusion [8, 11, 12], and the study subjects did not include children below the age of 10 years [9, 10]. A French study published in 1998 included a large number of hair samples (481) collected from age groups ranging from 2 to 83 years [13]. However, only 3.7% of this population consisted of children less than 10 years of age. The inconclusive nature of these studies originates primarily from the small study sample sizes, which often produce imprecise average value ranges with wide confidence intervals. To compensate for the large reference values in element concentrations in human hair, studies of larger samples sizes are needed to evaluate sex- and age-dependence.

In this study, we evaluated the sex- and age-dependence of major elements in scalp hair from 322 hair samples collected from participants aged 0–89 years, with approximately 20% of the samples collected from children less than 10 years of age.

## Methods

### Sample size and age distribution

To study the age- and sex- dependence of elements in human hair, the sample size was determined using the G power statistical program by incorporating an effect size of 0.25, significance level α of 0.05, and power of 0.8 with a group of 10. The calculated sample size was 260 subjects. We recruited 30% more hair donors than the calculated sample size. The subjects lived in the southern suburbs of Seoul (Hwaseong, Yongin, and Osan cities in Gyeonggi-do), South Korea. These intertwined cities, which include rural areas, were selected to represent the average Korean living environment. All participants gave informed consent or assent for this study and were compensated for their participation in all assessments. For minors, parental informed consent was obtained. The study was approved by the institutional review board of Eulji University (EUIRB 2017–48). A total of 322 hair samples were collected; the age- and sex-distributions of the hair sample donors are shown in Table 1. Samples were collected from 180 females (55.9%) and 142 males (44.1%) in the sample population.

**Table 1:** Age- and sex-distribution of hair sample donors

Age range (years)	Number of subjects (N)			Percent (%)
	Female	Male	Total	
0–4	14	14	28	8.70
5–9	11	28	39	12.11
10–14	15	17	32	9.94
15–19	7	9	16	4.97
20–29	21	8	29	9.01
30–39	24	10	34	10.56
40–49	35	16	51	15.84
50–59	19	9	28	8.70
60–69	7	9	16	4.97
70–79	11	17	28	8.70
> 80	16	5	21	6.52
Total	180	142	322	100.00

### Elemental analysis

Hair samples were collected from only healthy men and women who had not dyed, permed, or discolored their hair in the last 3 months. Hair was collected (5–10 cm) as close to the skin of the head as possible using surgical scissors. The hair samples were then cut into 50-mg slices and washed according to International Atomic Energy Agency recommendations [14]. Specifically, they were rinsed once in acetone, twice in double deionized water, and once more in acetone for 10 min each time [14]. Fifty-milligram portions of pretreated hair samples were measured and hydrolyzed for 30 min using nitric acid and hydrogen peroxide in the Mars 6 microwave digestion system (Matthews, NC, USA). The hydrolyzed homogeneous aqueous solutions were diluted and analyzed with an inductively coupled plasma mass spectrometer (Model X series II, Thermo Fisher Scientific, Waltham, MA, USA). All chemicals were of reagent grade and obtained from Sigma Aldrich (St. Louis, MO, USA).

Statistical analysis in this study, including correlation and linear regression analyses, were performed using Minitab® software (Minitab Inc., State College, PA, USA), and sex differences were analyzed using the paired *t*-test.

## Results

**Fig. 1:**
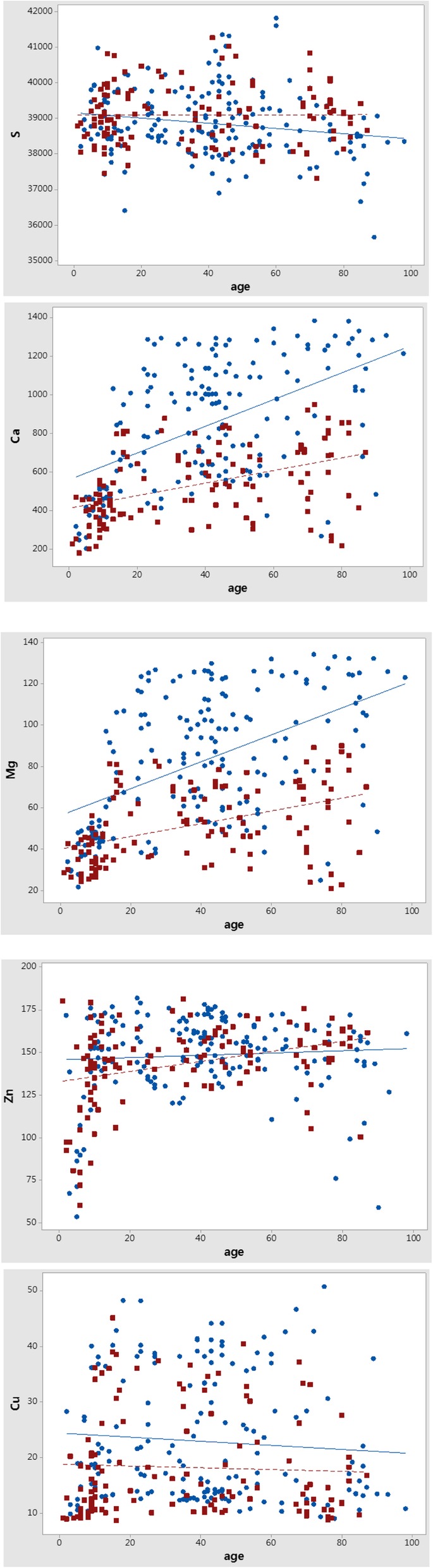
Age- and sex-dependence of 5 major elements found in scalp hair (μg/g of hair). Data from male samples are represented by squares, and data from female samples are indicated by circles. The solid line is the least squares fitting result of age-dependence for female subjects (solid line) and for male subjects (broken line). Calcium and magnesium concentrations exhibit strong age- and gender-dependencies, but this dependence is weak for sulfur, zinc, and copper

The concentrations of the five essential elements in human hair (sulfur, calcium, magnesium, zinc, and copper) are plotted against subject age in Fig. 1, where the circles represent element concentrations in samples from the female subjects and squares represent those from the male subjects. The age dependence of elements within male and female subjects was analyzed separately, and the solid line indicates the linear regression for the data from female subjects; the broken line represents those of the male subjects. The results of statistical analyses are presented in Table 2. The Pearson coefficient is a measure of the linear correlation between the element concentrations found in hair and age of the hair donor. This correlation shows a value between + 1 and − 1, where + 1 indicates a total positive linear correlation, 0 indicates no linear correlation, and − 1 is a total negative linear correlation. In linear regression analysis, S.E. represents the standard error of regression or absolute measure of the typical distance that the element concentration data falls from the regression line. R^2^ is the relative measure of the percentage of the element concentration variance that the model explains, ranging from 0 to 100%. A higher R-squared value indicates that the data points are closer to the fitted line.

**Table 2:** Statistical analysis of scalp hair element concentrations stratified on sex and age

		Correlation analysis		Linear regression analysis		
Element	Sex	Pearson coefficient	*p*-value	Equation	S. E.	R^2^, %
S	M	0.003	0.971	39,094 + 0.100*yr	814.8	0.0
	F	−0.181	0.019	39,160–7.331*yr	954.5	3.3
Ca	M	0.458	<0.001	412.1 + 3.272*yr	170.3	20.9
	F	0.521	<0.001	560.9 + 6.915*yr	270.9	27.2
Mg	M	0.447	<0.001	39.92 + 0.310*yr	16.62	20.0
	F	0.495	<0.001	56.33 + 0.651*yr	27.32	24.5
Zn	M	0.341	<0.001	132.9 + 0.297*yr	21.92	11.6
	F	0.068	0.378	145.7 + 0.067*yr	23.63	0.5
Cu	M	−0.045	0.614	18.86–0.016*yr	9.470	0.2
	F	−0.077	0.318	24.42–0.037*yr	11.29	0.6

S. E. indicates standard error

### Age-dependence of element concentrations in all age subjects

#### Sulfur

The linear regression for sulfur concentration relative to age is shown in Fig. 1 and suggests a slightly negative correlation between age and sulfur concentration in women (solid line) and no age dependence in men (broken line). The R^2^ value (3.3%) for the data from the female subgroup indicates that the age dependence in this subgroup is negligible, which is consistent with the data in males.

#### Calcium

Linear regression analysis for the calcium concentration relative to age is presented in Fig. 1 and suggests that calcium concentrations in female and male samples were strongly related to age. The slope of the regression line for female hair is two-fold greater than that of male hair. Based on this, we hypothesized that the calcium concentration in human hair increases with age in both men and women. However, the Pearson coefficient was approximately 0.5 and the R^2^ values for the linear regressions were 20.9 and 27.2% for men and women, respectively, indicating that only a small portion of the data can be explained by age dependence. Hair calcium concentrations show a large degree of individual variations because of differences in the environment, dietary intake, and health status, in addition to the age and sex, of people.

#### Magnesium

The age dependence of magnesium in human hair was very similar to that of calcium, as shown in Fig. 1. The magnesium concentration increased with age in both men and women. The age-dependence of magnesium was stronger in female hair than in male hair, similar to the results observed for calcium. Based on these findings, the magnesium concentration in human hair may increase with age in both men and women. However, only 20.0% of the male and 24.5% of the female data were explained by the linear relationship, indicating that individual differences also affected these samples.

#### Zinc

The linear regression analysis for the zinc concentration relative to age is presented in Fig. 1. The data suggest a slightly increasing trend in the male group, whereas the female grou*p* values remained nearly constant. However, the R^2^ value for the male subgroup indicates that only 11.6% of the data can be explained by the linear relationship.

#### Copper

The results of linear regression analysis of the copper concentration relative to age in Fig. 1 suggests a slightly decreasing trend in copper concentration with age for both male and female samples. The negative Pearson coefficients for copper presented in Table 2 confirm these results. However, small Pearson coefficients and nearly zero R^2^ values indicate that there is no correlation between these two variables.

### Age-dependence of element concentrations in young subjects

**Fig. 2:**
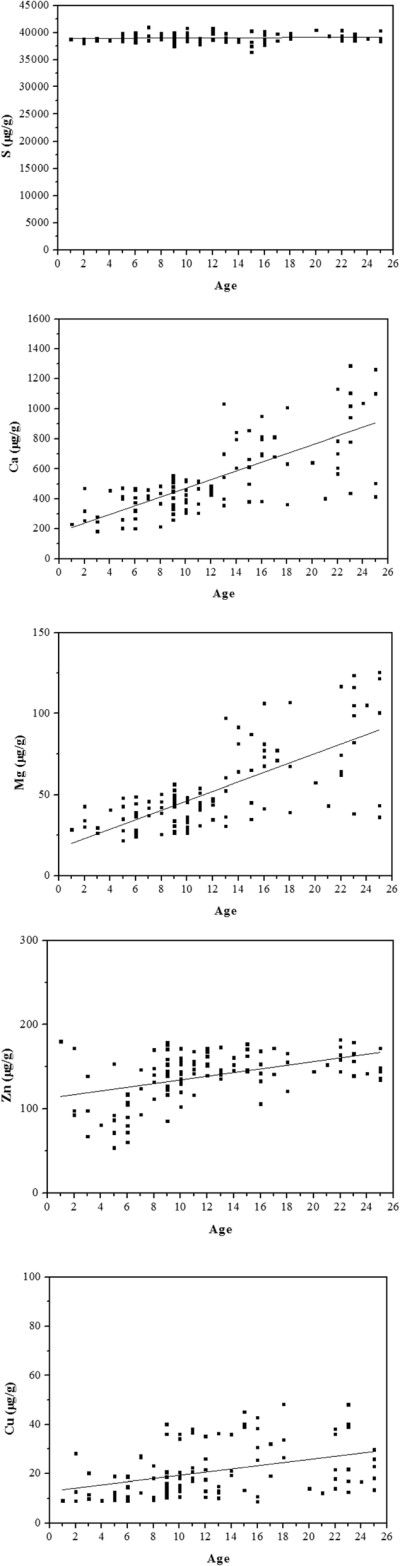
Age-dependence of 5 major elements found in scalp hair in subjects under 25. The male and female data are combined for the purpose of this analysis. The solid lines represent the linear least-squares regression results

The mineral element concentrations in human hair increased with age, particularly for calcium and magnesium in the lower age groups, and sex-dependent variation of elemental concentrations was observed only in older samples (Fig. 1). Based on these findings, we stratified the data based on age to evaluate participants under the age of 25 years independently. This under-25 years age group included both male and female subjects. The linear least-squares fitting results of the hair element concentrations for the under-25 years age group are shown in Table 3 and Fig. 2.

**Table 3:** Linear least squares fitting results for hair element concentrations in the under-25 years subgroup

Element	Intercept	Slope	R^2^, %
S	38,889 (±165)	10 (±12)	0.2
Ca	180 (±35.6)	29.1 (±2.6)	52.8
Mg	17.1 (±3.8)	2.92 (±0.28)	49.8
Zn	112.3 (±5.4)	2.19 (±0.40)	21.0
Cu	12.9 (±2.1)	0.64 (±0.15)	13.5

The fitting results indicated no age-dependence for sulfur concentration in hair, confirming the results in the larger sample population. Calcium and magnesium concentrations showed strong age-dependence in subjects under 25 years of age. The linear age-dependence explained nearly half of the differences in subjects up to 25 years of age, regardless of sex. We also observed age-dependence in zinc and copper accumulation, but the linear age-dependence explained only 21.0% and 13. 5% of the data, respectively.

### Sex-dependence of element concentrations in subjects of all ages

The linear regression results of elemental analysis of human hair obtained from both male and female participants are shown in Fig. 1 and indicate strong sex-dependence for both calcium and magnesium concentrations. We further analyzed the sex-dependence of calcium, magnesium, zinc, and copper concentrations in 11 different age groups. Children were divided into 4 age groups (0–4, 5–9, 10–14, and 15–19 years) and adults were divided into 7 age groups (20–29, 30–39, 40–49, 50–59, 60–69, 70–79, and 80–89 years).

#### Calcium

The average calcium concentration in female samples was larger than that in male samples in all age groups except for in the newborn age group (Table 4). Additionally, the average female hair calcium concentration was larger than that of males by more than 50% in all adult age groups, as shown by the F/M ratio in Table 4. The p-value of the  average calcium concentration between the sexes, F/M ratio, significantly decreased with age and was less than 0.01 in all subjects older than 30 years. Thus, the average calcium concentration in hair is at least 50% higher in adult females than in adult males.

**Table 4:** Sex-dependence of calcium concentration in human hair (μg/g hair)

Age (years)	Female			Male			F/M ratio	*p*-value
	Number	Average	S.D.	Number	Average	S.D.		
0–	3	283	36	5	319	135	0.89	0.6757
5–	14	426	97	22	383	98	1.11	0.2063
10–	15	551	181	17	427	119	1.29	0.0276
15–	7	758	185	9	617	192	1.23	0.0161
20–	19	892	289	7	580	209	1.54	0.0156
30–	23	894	246	10	573	158	1.56	0.0007
40–	36	931	239	16	607	150	1.53	<0.0001
50–	17	781	254	10	522	160	1.50	0.0078
60–	10	1066	239	7	674	74	1.58	0.0008
70–	10	1032	428	16	618	242	1.67	0.0042
80–	15	1101	247	8	649	253	1.70	0.0005

S.D. indicates standard deviation

F/M ratio is the ratio of the female average calcium concentration to the male average calcium concentration

#### Magnesium

The sex-dependence of the magnesium concentration in hair was similar to that observed for calcium (Table 5). The average magnesium concentration in female subjects was higher than that in male subjects in all age groups except for in newborns. Additionally, the average magnesium concentration in female samples was larger than that in males by more than 40% in all adult age groups except for the 50–59-year-old group, as shown by the F/M ratio in Table 5. Differences in the average calcium concentration between sexes were significant (*p* < 0.005) in all age groups over 30 years of age, except for in the 50–59 years age group. The reason for the aberrantly low F/M ratio and high *p*-value observed in this subgroup for magnesium requires further analysis.

**Table 5:** Sex-dependence of magnesium concentration in human hair (μg/g hair)

Age (years)	Female			Male			F/M ratio	*p*-value
	Number	Average	S.D.	Number	Average	S.D.		
0–	3	31.1	2.6	5	33.7	7.5	0.92	0.6757
5–	14	41.6	9.1	22	33.6	9.3	1.24	0.2063
10–	15	53.5	18.5	17	39.4	13.5	1.36	0.0276
15–	7	82.5	21.8	9	61.2	17.7	1.35	0.0161
20–	19	88.0	33.2	7	56.8	18.2	1.55	0.0279
30–	23	85.5	22.1	10	59.9	9.2	1.43	0.0014
40–	36	92.9	25.0	16	55.0	14.6	1.69	<0.0001
50–	17	75.8	26.2	10	63.5	11.9	1.19	0.1756
60–	10	102.9	23.7	7	64.8	9.5	1.59	0.0012
70–	10	99.4	42.8	16	56.8	23.6	1.75	0.0031
80–	15	108.1	20.7	8	65.7	24.7	1.65	0.0003

S.D. indicates standard deviation

F/M ratio is the ratio of the female average calcium concentration to the male average calcium concentration

#### Zinc

The linear regression results for zinc concentration in human hair in male and female subgroups (shown in Fig. 1) suggest low sex-dependence in the zinc concentration. The comparison of male and female zinc concentrations in various age groups (Table 6) revealed that the difference in zinc concentrations between the male and female subgroups was negligible when the number of subjects in the age group was large enough.

**Table 6:** Sex-dependence of zinc concentration in human hair (μg/g hair)

Age (years)	Female			Male			F/M ratio	*p* value
	Number	Average	S.D.	Number	Average	S.D.		
0–	3	126.0	53.0	5	110.0	40.0	1.15	0.6418
5–	14	116.0	38.0	22	128.0	32.0	0.91	0.3150
10–	15	153.0	12.0	17	143.0	19.0	1.07	0.0901
15–	7	160.0	16.0	9	143.0	20.0	1.12	0.0880
20–	19	151.0	15.1	7	149.0	7.6	1.01	0.9973
30–	23	153.6	18.9	10	155.2	17.5	0.99	0.5361
40–	36	156.7	14.5	16	150.5	11.9	1.04	0.1533
50–	17	153.9	9.0	10	157.4	16.0	0.98	0.5112
60–	10	151.4	21.5	7	158.0	11.0	0.96	0.4523
70–	10	142.0	24.6	16	146.8	16.2	0.97	0.5381
80–	15	139.3	30.0	8	152.8	22.1	0.91	0.2595

S.D. indicates standard deviation

F/M ratio is the ratio of the female average calcium concentration to the male average calcium concentration

#### Copper

The linear regression results for copper concentration in human hair in the male and female subgroups (shown in Fig. 1) suggests that the average copper concentration in the female subgroup was higher than that in the male subgroup for all ages. The average copper concentration of the male subgroup (18.55 ± 4.65, *n* = 142) significantly differed from that of the female subgroup according to the *t*-test (*p*-value < 0.0001).

This sex-dependence could not be confirmed when the average copper concentrations in the female and male subgroups in specific age groups were compared. The average copper concentration in females was larger than that in males in eight of the 11 age groups, effectively the same in one group, and smaller in two age groups. Additionally, the sex-dependence of copper concentration in human hair was not significant when the subject number was small (*p* > 0.05) (Table 7).

**Table 7:** Sex-dependence of copper concentration in human hair (μg/g hair)

Age (years)	Female			Male			F/M ratio	*p*-value
	Number	Average	S.D.	Number	Average	S.D.		
0–	3	16.6	10.2	5	12.1	4.8	1.37	0.4172
5–	14	19.6	9.6	22	13.8	4.5	1.42	0.0194
10–	15	23.2	9	17	19.8	9.8	1.17	0.3172
15–	7	33.1	14	9	27.9	12.7	1.19	0.4535
20–	19	26.7	11.5	7	20.7	12.5	1.29	0.2598
30–	23	21.1	10.0	10	22.5	10.7	0.94	0.7198
40–	36	24.1	12.1	16	17.5	9.5	1.38	0.0593
50–	17	22.5	10.0	10	25.9	8.5	0.87	0.3771
60–	10	27.6	14.6	7	21.7	10.8	1.27	0.3792
70–	10	21.6	16.4	16	12.5	5.6	1.72	0.0507
80–	15	16.6	5.4	8	16.5	5.5	1.00	0.9669

S.D. indicates standard deviation

F/M ratio is the ratio of the female average calcium concentration to the male average calcium concentration

## Discussion

The average sulfur concentrations in hair were 39,068 ± 443 and 38,727 ± 402 μg/g in the male and female subgroups, respectively. These values were larger than those reported in a Polish study (33,810 ± 1994 μg/g), but smaller than those reported in a Swedish study (47,700 ± 5400 μg/g) [15] and Japanese study (43,300 ± 6400 μg/g) [15–17]. This suggests that additional studies are needed to determine if these differences result from ethnic differences in the study populations.

Calcium and magnesium hair concentrations were found to be significantly sex-dependent in the Swedish study [15], with calcium concentrations in scalp hair of 461 ± 309 μg/g in men and 1040 ± 860 μg/g in women; the magnesium concentrations were 31 ± 20 μg/g in men and 56 ± 42 μg/g in women. They also found no significant differences in sulfur, zinc, and copper concentrations in human scalp hair between the two sexes. These results agree with those of our study in terms of the sex dependence of the concentrations of major elements in human scalp hair.

We demonstrated that the calcium and magnesium concentrations in scalp hair were strongly sex-dependent, particularly in adults over 20 years of age. It is, however, difficult to demonstrate age dependence of element concentrations in scalp hair if the results are not stratified for sex or if the number of samples collected per age group is not large enough. We found that the concentrations of calcium, magnesium, zinc, and copper increased with age in younger subjects (those under 25 years old), as a large number of samples was collected from each age subgroup. Combining this data with the already established concept of bone peak density suggests that mineral element concentrations increase with age in scalp hair, as observed in bone, in younger people.

## Conclusions

We observed significant age- and sex-dependence in the concentrations of major mineral elements in human scalp hair. Increases in the concentrations of calcium, magnesium, zinc, and copper in the scalp hair samples from people up to 25-years-old were significant. Strong sex dependence for both calcium and magnesium concentrations in scalp hair was also detected in study participants over the age of 20 years; both calcium and magnesium were significantly more concentrated in female participants. The concentrations of sulfur, zinc, and copper showed no significant sex-dependence.

## Data Availability

For data requests, please contact the corresponding author.

## Abbreviations

ICP MS: Inductively coupled plasma mass spectrometry

## References

[1] Verma A, Singh VK, Verma SK, Sharma A (2016). Human hair: a biodegradable composite fiber–a review. Int J Waste Resour.

[2] Popescu C, Höcker H (2007). Hair - the most sophisticated biological composite material. Chem Soc Rev.

[3] Mahdavi-Roshan M, Ebrahimi M, Ebrahimi A (2015). Copper, magnesium, zinc and calcium status in osteopenic and osteoporotic post-menopausal women. Clin Cases Miner Bone Metab.

[4] Miekeley N, de Fortes Carvalho LM, Porto da Silveira CL (2001). Elemental anomalies in hair as indicators of endocrinologic pathologies and deficiencies in calcium and bone metabolism. J Trace Elem Med Biol.

[5] MacPherson A, Bacsó J (2000). Relationship of hair calcium concentration to incidence of coronary heart disease. Sci Total Environ.

[6] Park SJ, Lee SH, Cho DY, Kim KM (2013). Hair calcium concentration is associated with calcium intake and bone mineral density. Int J Vitam Nutr Res.

[7] Xue Shanshan, Kemal Oumer, Lu Meihan, Lix Lisa M., Leslie William D., Yang Shuman (2020). Age at attainment of peak bone mineral density and its associated factors: The National Health and Nutrition Examination Survey 2005–2014. Bone.

[8] Sturaro A, Parvoli G, Doretti L, Allegri G, Costa C (1994). The influence of color, age, and sex on the content of zinc, copper, nickel, manganese, and lead in human hair. Biol Trace Elem Res.

[9] Tamburo E, Varrica D, Dongarrà D (2016). Gender as a key factor in trace metal and metalloid content of human scalp hair. A multi-site study. Sci Total Environ.

[10] Skalnaya MG, Tinkov AA, Demidov VA, Serebryansky EP, Nikonorov AA, Skalny AV (2016). Age-related differences in hair trace elements: a cross-sectional study in Orenburg. Russia Ann Hum Biol.

[11] Zhu Y, Wang YZ, Meng FJ, Zhang H, Ren Q, Shen H (2018). Distribution of metal and metalloid elements in human scalp hair in Taiyuan. China Ecotoxicol Environ Saf.

[12] Skalny AV, Simashkova NV, Skalnaya AA, Klyushnik TP, Bjørklund G, Skalnaya MG (2017). Assessment of gender and age effects on serum and hair trace element levels in children with autism spectrum disorder. Metab Brain Dis.

[13] Zakrgynska-Fontaine V, Dore JC, Ojasoo T, Poirier-Duchene F, Viel C (1998). Study of the age and sex dependence of trace elements in hair by correspondence analysis. Biol Trace Elem Res.

[14] Ryabukhin YS. Activation analysis of hair as an indicator of contamination of man by environmental trace element pollutants. Vienna: International Atomic Energy Agency. Report no. IAEA/RL/50. 1978. Vienna.

[15] Rodushkina IU, Axelsson MD (2000). Application of double focusing sector field ICP-MS for multielemental characterization of human hair and nails. Part II A study of the inhabitants of northern Sweden. Sci Total Environ.

[16] Baranowska I, Barchański L, Bąk M (2004). X-ray fluorescence spectrometry in multielemental analysis of hair and teeth. Pol J Environ Stud.

[17] Sera S, Futatsugawa S, Murao S (2002). Quantitative analysis of untreated hair samples for monitoring human exposure to heavy metals. Nucl Instrum Methods Phys Res B Beam Interact Mater At.

